# Structure of TeO_2_ Glass and Melt by Reverse Monte Carlo Simulations of High-Energy X-Ray Diffraction Data Sets

**DOI:** 10.1021/acsomega.4c02425

**Published:** 2024-05-07

**Authors:** Atul Khanna, Margit Fábián

**Affiliations:** † Sensors and Glass Physics Laboratory, Department of Physics, Guru Nanak Dev University, Amritsar, Punjab 143005, India; ‡ HUN-REN Centre for Energy Research, Budapest 1121, Hungary

## Abstract

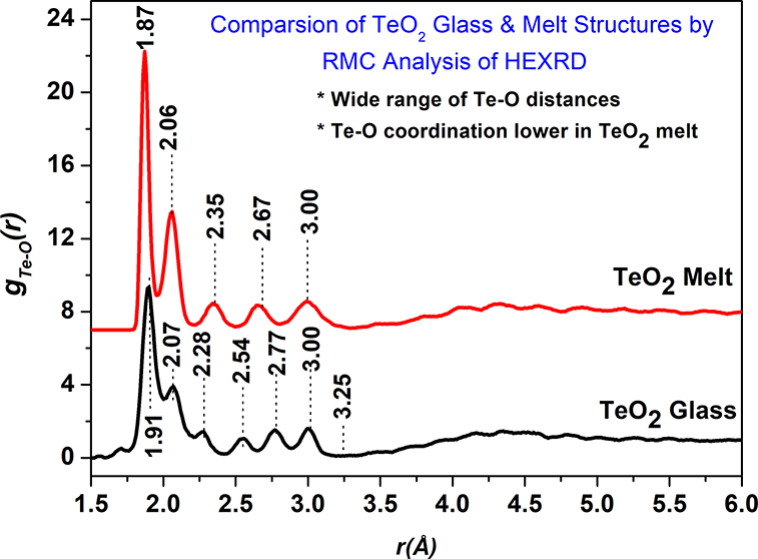

The short-range and medium-range structures of TeO_2_ glass and melt are elucidated by Reverse Monte Carlo (RMC) simulations of High-Energy X-ray Diffraction data sets published in an earlier study by Alderman et al. (*J. Phys. Chem. Lett.***11**(1) (2020)427–431). The RMC analysis reveals that there exists a wide range of Te-O bond lengths in both TeO_2_ glass and melt short-range structures. The Te-O pair distribution function (PDF) of the melt has peaks centered at 1.87, 2.06, 2.35, 2.65, and 3.00 (±0.01) Å, whereas the corresponding peaks in the glass are at 1.91, 2.07, 2.28, 2.54, 2.77, and 3.00 (±0.01) Å. The Te-O partial PDF of the melt shows a peak at 2.35 Å, which is not present in the glass structure; therefore, the same co-ordination sphere radius of 2.36 Å cannot be used for calculating the Te-O co-ordination numbers in the TeO_2_ melt and glass, as done in the earlier study by Alderman et al. Using a more appropriate radius of 2.41 Å for glass and 2.22 Å for the melt, the corresponding Te-O co-ordination numbers are found to be 3.99 and 3.33, respectively. The RMC analysis successfully determined the O-O pair distributions, which show the first peaks at 2.31–2.33 (±0.01) Å. Finally, Te-Te pair distributions show peaks at slightly longer distances in the melt compared to those in glass, and the melt is found to have greater medium-range disorder.

## 1 Introduction

TeO_2_ is a conditional glass former; it forms a glassy phase at high melt-quenching rates of ∼10^4^–10^5^ K·s^–1^ by a twin roller quenching technique. The short-range structure of TeO_2_ glass was studied by Barney et al. and Te-O co-ordination number of 3.68 was reported by high-*Q* neutron diffraction studies.^1^ Gulenko et al. reported Te-O co-ordination number of 3.73 in TeO_2_ glass.^2^ It is well-known that the glass-forming ability of TeO_2_ enhances drastically; and the Te-O co-ordination number decreases with the addition of alkali, alkaline-earth, and heavy metal oxides in the tellurite network.^3−5^ Tellurite glasses and crystals are technologically important materials and have applications in optical fibers, Raman amplifiers, and nonlinear optical devices.^6^ It is important from a fundamental point of view to understand the relationship between a liquid and its glass and compare the Te-O co-ordination numbers in molten and glassy TeO_2_.

Alderman et al. carried out High-Energy X-ray Diffraction (HEXRD) studies of TeO_2_ glass and melt using the containerless levitation technique and determined the total pair correlation distributions, *T(r)*, by Fourier transformation of the X-ray diffraction structure factors. Alderman et al. found short-range disorder due to the existence of wide distribution of Te-O bond lengths/distances in the glass and melt structures.^7^ These investigators pointed out the uncertainty in the calculation of Te-O co-ordination number in TeO_2_ glass and melt due to the difficulty in selecting the radius of the first Te-O co-ordination sphere from *T(r)* distributions. The *T(r)* distributions of TeO_2_ glass and melt have very similar shapes, and Alderman et al. used a radius of 2.36 Å to calculate the Te-O co-ordination numbers. It was concluded from this study that Te-O co-ordination number is 4.22 in TeO_2_ glass and only a slightly lower value of 4.09 (±0.22) in the TeO_2_ melt.^7^

The earlier investigators used the same co-ordination sphere radius value of 2.36 Å for calculating the Te-O co-ordination number in both glass and melt samples.^7^ The use of the same value of *r*_max_ = 2.36 Å for both glass and melt is not valid if any peak exists at this position in Te-O atomic pair correlations. It is important to determine the partial pair distributions of Te-O atomic pairs to clearly understand the distributions in Te-O bond lengths and correctly determine the first co-ordination sphere radius and hence the Te-O co-ordination numbers. The total pair distribution function, *T(r),* calculated by the Fourier sine transformation of structure factors by Alderman et al. contains overlapping pair distributions of Te-O and O-O atomic pairs at approximately the same distances, and hence, it is difficult to calculate the Te-O co-ordination numbers unambiguously, especially when a comparison is to be made of Te-O bond lengths and co-ordination numbers in glassy and molten TeO_2_.

The present study reports the results of Reverse Monte Carlo (RMC) simulation studies on HEXRD data sets published in an earlier study.^7^ The partial Te-O, O-O, and Te-Te atomic pair distributions were calculated by the RMC technique and used to elucidate the TeO_2_ glass and melt short-range structural properties, and a comparison of Te-O co-ordination numbers, short-range, and medium- range order was carried out in glassy and molten states of TeO_2_.

## 2 Experimental Methods

The High-Energy X-ray Diffraction data sets of TeO_2_ glass and melt, measured earlier by Alderman et al. These are reported in the supplementary file of their article,^7^ and have been used in the present study for simulations using RMC++ software^8^ to generate the partial pair correlation functions and determine the Te-O coordination numbers in both glassy and molten TeO_2_.

The RMC simulation method is an effective tool for building 3D structural models that are consistent with the experimental data and total structural factors obtained from X-ray diffraction experiments. During the RMC analysis, the difference between the experimental and calculated structural factors is minimized by random movement of the particles. At the end of the simulation, a particle configuration is obtained. From these final configurations, structural properties, i.e., partial pair correlation functions of Te-O, O-O, and Te-Te and the Te-O co-ordination numbers can be calculated.^8^

In a sample, such as TeO_2_, the total number of atoms in each molecule, *k* = 2, and hence *k(k*+1)*/*2 = 3 atomic pairs, i.e., Te-Te, Te-O, and O-O exist with different X-ray scattering amplitudes that are strongly *Q*-dependent. The weight factor values, *w*_*ij*_ for the *ij*^th^ atomic pairs were calculated using the following formula:
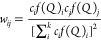
1where *f*_*i*_(*Q*) and *f*_*j*_(*Q*) are the X-ray scattering amplitudes of the *i*^th^ and *j*^th^ atoms in the sample, respectively. Table 1 gives the values of *w*_ij_ (at *Q* = 2.51 Å^–1^) for the three atom pairs in the TeO_2_ glass and melt samples. The RMC simulation calculates the one-dimensional partial atomic pair correlation functions, *g*_*ij*_(*r*), which are Fourier sine transformed to calculate the partial structure factors, *S*_ij_(*Q*). The disordered atomic configuration was first built up to run the RMC program with a simulation box that contained 10000 randomly distributed atoms of Te and O. The atomic number density values of 0.064 Å^–3^ and 0.057 Å^–3^ were used for the TeO_2_ glass and melt samples, respectively.^7^

**Table 1:** Values of Minimum Distances, *r*_cutoff_ (Å), used in the Input file of the RMC Program for Different Atomic Pairs in the TeO_2_ Glass and Melt Samples^a^

atomic pair	TeO_2_ glass	TeO_2_ melt
Te-Te (61.5%)	3.05	3.10
Te-O (33.8%)	1.55	1.60
O-O (4.7%)	2.20	2.25

a The X-ray scattering weight factors (in %) for aach atomic pair at *Q* = 2.51 Å^–1^ are also given.

During the RMC simulations, the minimum interatomic distances were used as constraints to fit the model with experimental value *S*(*Q*). However, no constraints were imposed for Te-O coordination in the RMC input program. Repeated RMC runs were performed by modifying the minimum distance values slightly in such a way to produce reliable data for each partial pair correlation function, *g*_*ij*_(*r*). The final minimum distance between the three atomic pairs (*r*_cutoff_) values used in the RMC input program are given in Table 1. The different *r*_min_ and *r*_max_ values used to calculate the corresponding Te-O co-ordination numbers are presented in Table 2.

**Table 2:** Te-O Co-ordination Numbers in the TeO_2_ Melt and Glass Samples in Different Ranges of *r*_min_ and *r*_max_ (Å, in Brackets)

Te-O co-ordination in melt (±0.01)	Te-O co-ordination in glass (±0.01)
3.33 (1.50–2.22)	3.53 (1.50–2.22)
3.33 (1.50–2.22)	3.99 (1.50–2.41)
3.65 (1.50–2.36)	3.95 (1.50–2.36)
3.90 (1.50–2.52)	4.65 (1.50–2.81)
6.14 (1.50–3.25)	6.56 (1.50–3.25)

**Figure 1:**
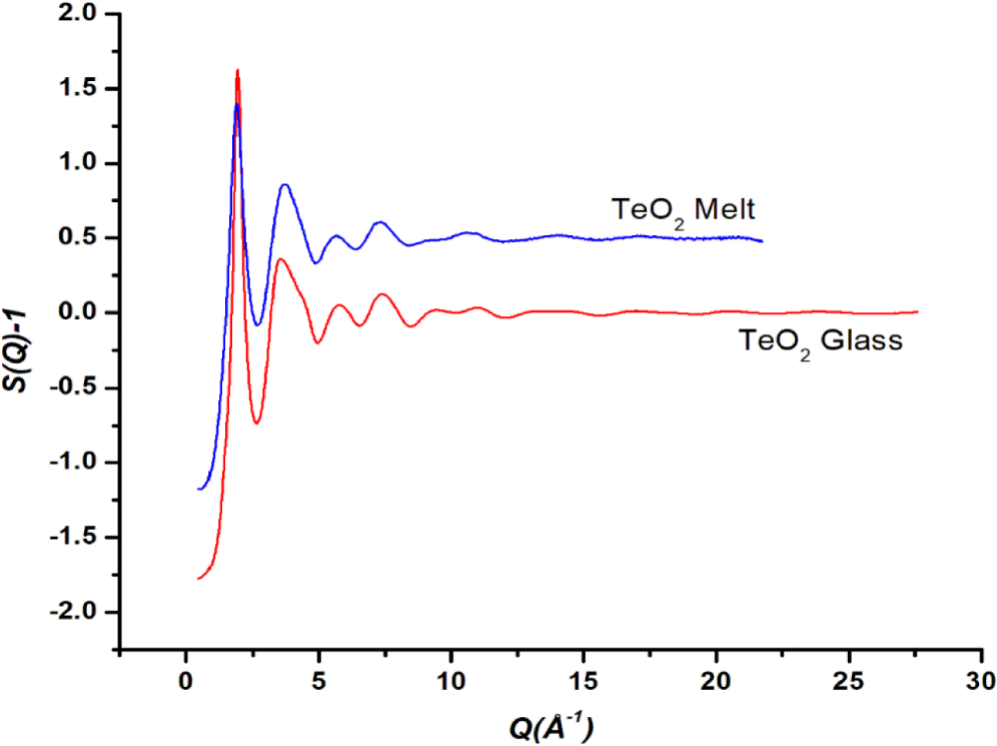
Experimental and RMC-calculated total X-ray structure factors of TeO_2_ glass and melt. RMC-calculated structure factors are in black and overlap perfectly with experimental curves in blue (melt) and red (glass). The curves for the melt sample are displaced by 0.5 unit for the sake of clarity.

The RMC simulations achieved perfect matching of the experimental and the calculated X-ray structure factors (Figure 1), and runs were repeated several times to check the reproducibility of the calculated Te-O, O-O, and Te-Te partial pair distributions and Te-O co-ordination environments.

## 3 Results and Discussion

**Figure 2:**
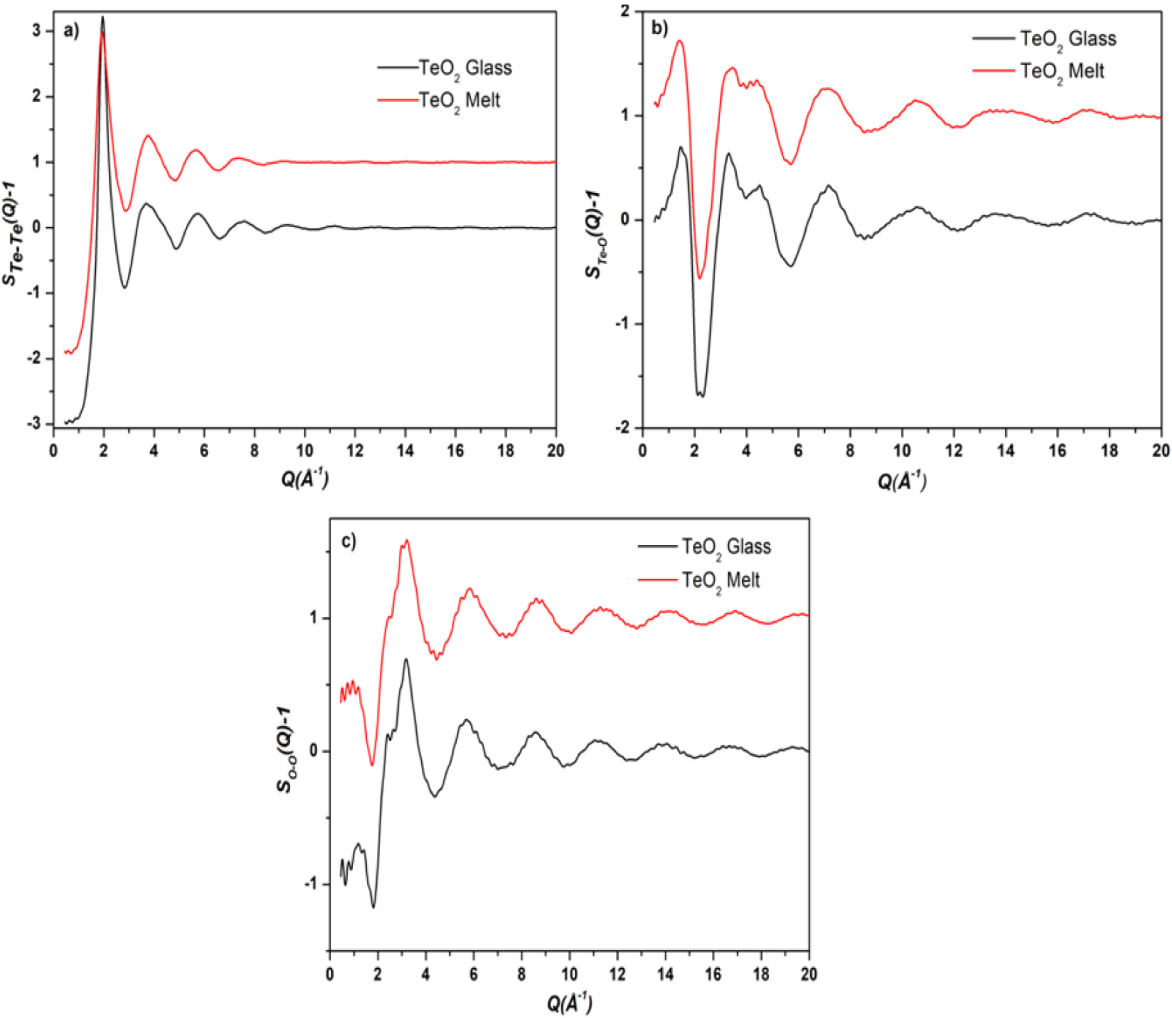
Partial structure factors of TeO_2_ glass and melt: (a) Te-Te, (b) Te-O, and (c) O-O. The curves for the melt sample are displaced by 1 unit for clarity.

Figure 1 shows the experimental and RMC-calculated total structure factors (*S*(*Q*)-1) of TeO_2_ glass and melt. The perfect overlap of the two structure factors for the glass and melt samples confirmed the success of the RMC technique. The partial structure factors (*S*_Te*-*Te_(*Q*)-1), (*S*_Te-O_(*Q*)-1), and (*S*_*O-O*_(*Q*)-1) in the two samples are displayed in Figure 2a-c, respectively. On comparing the three partial structure curves are compared with the total structure factor curves, it is clear that the Te-Te partial structure factor curves (Figure 2a) closely resemble the curves for total structure factors (Figure 1), which confirms that the FSDP at 1.8–1.94 Å^–1^ is mostly due to medium-range ordering of Te-Te atomic pairs, and the contribution of the O-O pair correlations in the FSDP is very small due to low scattering of X-rays by the O atoms. It should be noted that the weight factor for X-ray scattering by Te-Te pairs is maximum (66.7% at *Q* = 2.51Å^–1^) while that of the O-O pairs is smallest (4.7% at *Q* = 2.51 Å^–1^). The FSDP has a greater width in the TeO_2_ melt as compared to its width in the glass sample.

**Figure 3:**
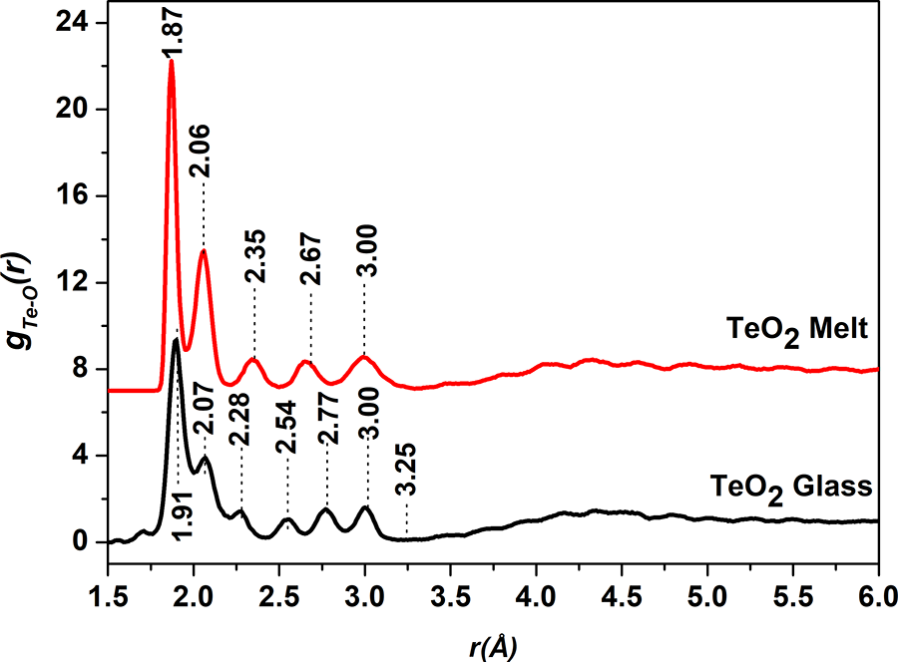
Te-O pair distribution functions in the TeO_2_ glass and melt structures were determined by RMC simulations. The partial pair distribution function of melt is displaced upward by 7 units for clarity.

**Figure 4:**
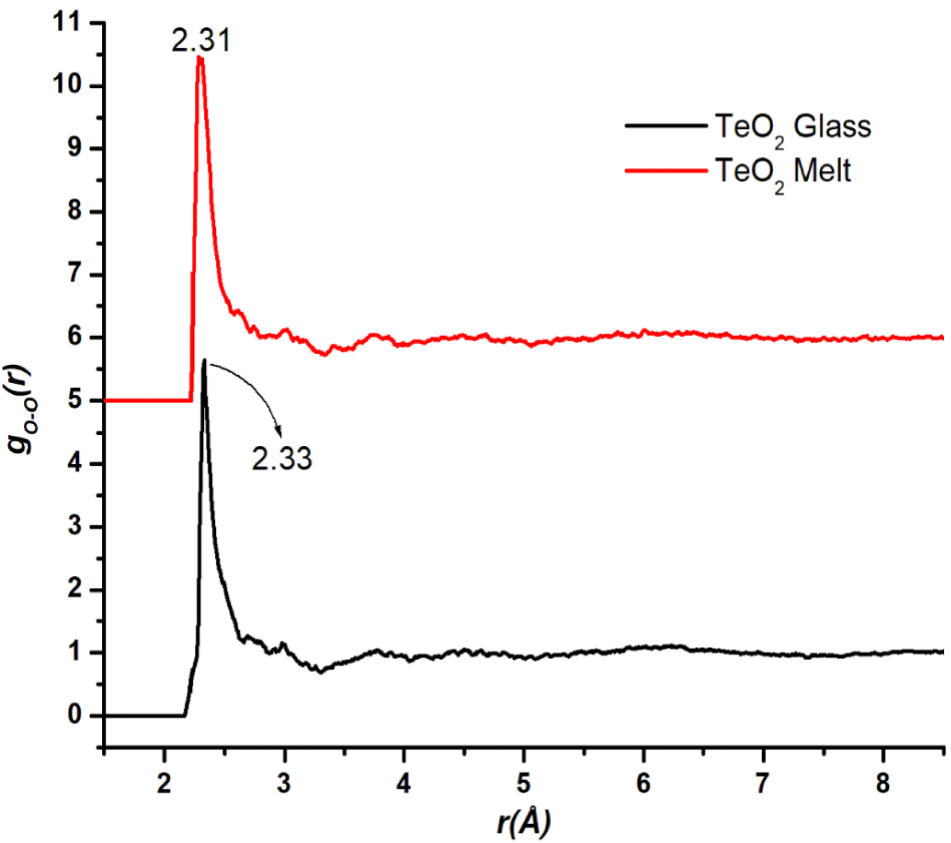
O-O pair distribution functions in the TeO_2_ glass and melt structures. The partial pair distribution function of the melt is displaced upward by 5 units for clarity.

**Figure 5:**
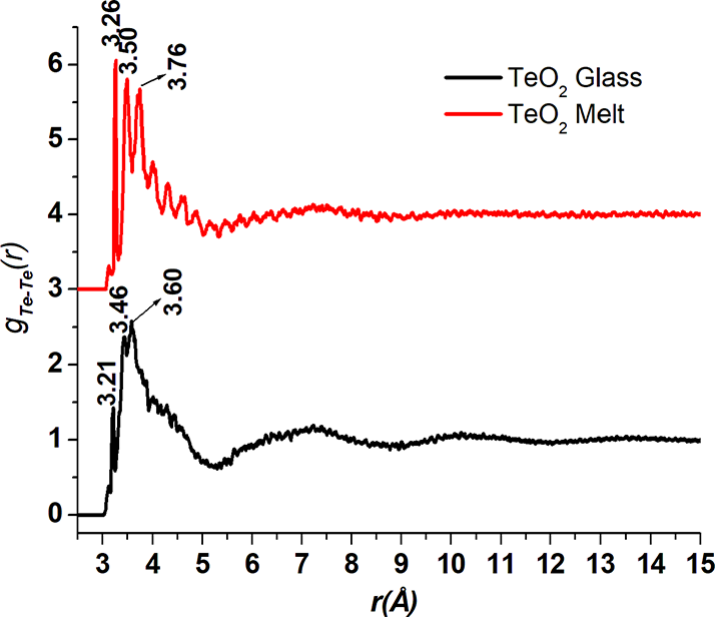
Te-Te pair distribution functions in the TeO_2_ glass and melt. The curve for the melt sample is displaced upward by 3 units for clarity.

The Te-O, O-O, and Te-Te atomic pair distributions for the glass and melt produced by the final RMC analysis are shown in Figure 3–5, respectively.

The Te-O atomic pair distributions for glass and melt calculated by the RMC technique is displayed in Figure 3. It is found that both TeO_2_ glass and melt short-range structures consist of a wide distribution of Te-O bond lengths/distances. The first peak in Te-O distribution of the melt occurs at 1.87 (±0.01) Å. The other Te-O peaks in the melt structure exist at 2.06, 2.35, 2.66, and 3.00 Å. In the case of TeO_2_ glass, the first Te-O peak is centered at a slightly longer distance of 1.91 Å, while the other peaks occur at 2.07, 2.28, 2.54, 2.77, and 3.00 Å. Interestingly, the first co-ordination shell of Te-O atomic pairs terminates at the same radius of 3.25 Å in both the glass and melt, and the last Te-O peak in both the glass and melt structures is centered at the same position of 3.00 Å. Further, the first two Te-O peaks at shorter distances of 1.87 and 2.06 Å in the melt structure are sharper and are more intense than the corresponding peaks at 1.91 and 2.07 Å in the glass sample. Therefore, the melt structure consists of a greater number of shorter Te-O bonds. On the other hand, the peaks at longer distances of 2.35, 2.67, and 3.00 Å are broader in the melt structure.

In several previous studies on the structural characterization of TeO_2_ glass and melt, an *r*_max_ radius of 2.35 Å has been used to calculate the Te-O co-ordination number in the TeO_2_ glass.^1,2,7^ The RMC analysis of HEXRD data revealed that there exists a fine structure in Te-O bond lengths/distances and a definite Te-O peak centered at 2.35 Å exists in the melt structure; therefore, this value of *r_max_* cannot be used to calculate Te-O co-ordination number at least in the case of the TeO_2_ melt.

A more reasonable value of *r*_max_ would be 2.22 Å in the melt, and if the same value is also used for the TeO_2_ glass sample, the Te-O co-ordination number is found to be 3.33 in the TeO_2_ melt and 3.53 in the TeO_2_ glass. The *r*_max_ value of 2.22 Å is valid because up to this radius, the first two peaks are unambiguously included in the Te-O pair correlations of both the TeO_2_ glass and the melt. However, if we include the third peak at 2.28 Å of TeO_2_ glass structure, then an *r*_max_ radius of 2.41 Å must be used, and in this case Te-O co-ordination number comes out to be 3.99. If we use the same radius of 2.36 Å for both glass and the melt as done by Alderman et al. the Te-O co-ordination number is found to be 3.95 in glass and 3.65 in the melt.^7^

To include the third peak at 2.35 Å of TeO_2_ melt structure, an *r*_max_ of 2.52 Å has to be used, and the corresponding Te-O co-ordination number in the melt is 3.90, which is again less than the corresponding value of 3.99 in the glass sample. On including the fourth peak at 2.67 Å and using an *r*_max_ radius of 2.81 Å, Te-O co-ordination in the melt is found to be 4.65, similarly, by using an *r*_max_ of 2.88 Å and by including the fifth peak at 2.77 Å in glass structure, Te-O co-ordination number is found to be 5.44. Finally, by including the last peak centered at 3.00 Å in both the TeO_2_ glass and melt and using an *r*_max_ of 3.25 Å (radius at which fine structure of Te-O pair correlations ends in the first co-ordination sphere of both the glass and melt samples), the Te-O co-ordination has values of 6.14 and 6.56 in TeO_2_ melt and glass, respectively. The earliest X-ray structural study of TeO_2_ glass containing a small amount of Li_2_O (1.84% by weight) was carried out by Brady, who reported that the Te-O co-ordination be 6 (octahedral) due to 4 oxygens surrounding each Te atom at shorter distances of 2.05, 2.07, 2.12 and 2.20 Å and another two O atoms at longer distances of 2.68 and 2.78 Å.^9,10^

It should be noted that the peaks at longer distances of 3.00 Å may be due to nonbonded Te-O atomic pairs. Therefore, it is concluded that Te-O co-ordination number is significantly lower in TeO_2_ melt as compared to that in TeO_2_ glass for all possible *r*_max_ radii. Table 2 shows the values of Te-O co-ordination numbers in glass and melt samples for different values of *r*_max_. It is concluded from the RMC analysis of the HEXRD data sets that first, the same *r*_max_ values cannot be used for determining the Te-O co-ordination numbers in TeO_2_ melt and glass, and second, the melt has a significantly lower Te-O co-ordination number compared to that in glass for the same *r*_max_ radius. The decrease of Te-O co-ordination number with an increase in temperature can be explained by the following isomerization reaction by virtue of which TeO_4_ structural units transform into TeO_3_ units with the simultaneous formation of nonbridging oxygen (NBO) in the structure:
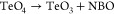
2

The above isomerization reaction proceeds forward at a greater rate with an increase in temperature and it is reported to influence the glass-forming ability of lead tellurite and strontium tellurite melts.^11^ Clear and conclusive evidence for the temperature-induced conversion of TeO_4_ into TeO_3_ units was provided by *in situ* high-temperature Raman studies on glass, supercooled and melts of TeO_2_.^12^ Similar isomerization reactions in which the tetrahedral BO_4_ units breakdown into BO_3_ units:
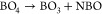
3occur in borate melts as a function of temperature and time and produce a large decrease in the B-O co-ordination numbers with an increase in temperature.^13−16^

The O-O partial pair distributions in the glass and melt samples are shown in Figure 4. These curves show the first peak at 2.33(±0.01) and 2.31(±0.01) Å in glass and melt structures, respectively. The RMC analysis revealed that O-O atomic pairs have the first peak at ∼2.3 Å, a distance that has been used in several earlier studies to calculate Te-O co-ordination number from total radial distribution function analysis.^1,2,7^ Hence, it is important to determine the partial pair distributions to correctly calculate the Te-O co-ordination numbers, and an *r*_max_ value of 2.35 Å cannot be used to calculate Te-O co-ordination from the total radial distribution function, as the latter will contain contributions of O-O correlations at approximately the same distance. The O-O pair distribution is broader in the melt structure, which indicates a more short-range O-O disorder in the melt (Figure 4).

The Te-Te pair distributions for the glass and melt (Figure 5) reveal that the Te-Te separations are greater in the melt as compared to those in the glass sample. This is indicated by the displacement of First Sharp Diffraction Peak (FSDP) of the total X-ray structure factors, which is positioned at lower *Q*-values in the melt (Figure 1). As discussed above, the FSDP is mostly due to medium-range ordering of Te-Te atomic pairs, and the contribution of O-O pair correlations in the FSDP is very small due to low scattering of X-rays by the O atoms. The FSDP is positioned at 1.88 Å^–1^ in the melt and at 1.94 Å^–1^ in the glass sample. The shifting of FSDP toward lower *Q* indicates greater atomic separations in the real space, and this is confirmed by the RMC results. The Te-Te pair distribution function shows the first three peaks at 3.21 ± 0.01, 3.46 ± 0.01, and 3.60 ± 0.01 Å in the glass sample and at 3.26 ± 0.01, 3.50 ± 0.01, and 3.76 ± 0.01 Å in the melt, respectively. Further, the Te-Te PDF contains several closely spaced peaks indicating higher medium-range disorder in the melt as compared to those in the glass sample. The amplitude of oscillations in Te-Te PDF is significantly greater at *r* > 6 Å in the glass as compared to that in the melt, which shows that a greater medium-range order exists in the glass structure.

## 4 Conclusions

It is concluded from the RMC analysis of the HEXRD data sets that Te-O co-ordination number in the TeO_2_ melt is significantly lower than that in the TeO_2_ glass, and the same value of *r*_max_ = 2.36Å cannot be used for calculating the Te-O co-ordination numbers in the glassy and molten TeO_2_. The decrease in the Te-O coordination number in the melt is attributed to the isomerization reaction: TeO_4_ → TeO_3_ + NBO, which proceeds rapidly toward forward direction at high temperatures. The RMC studies reveal fine structure in Te-O atomic pair correlations and the existence of a wide range of Te-O atomic pair correlations in both glassy and molten TeO_2_. The O-O atomic pair correlations were successfully calculated by the RMC technique and show peak at 2.31–2.33 Å. Finally, the Te-Te separations are larger and a greater medium-range disorder exists in the melt structure as compared to that in the glass structure.
